# In search of justification for the unpredictability paradox

**DOI:** 10.1186/1745-6215-15-480

**Published:** 2014-12-10

**Authors:** Jeremy Howick, Alexander Mebius

**Affiliations:** Department of Primary Care Health Sciences, University of Oxford, New Radcliffe House, 2nd floor, Oxford, OX2 6NW UK; Department of Philosophy and History of Technology, Royal Institute of Technology (KTH), Teknikringen 78 B, 100 44 Stockholm, Sweden

**Keywords:** Random allocation, Randomized controlled trial, Meta-analysis, Evidence-based medicine

## Abstract

A 2011 Cochrane Review found that adequately randomized trials sometimes revealed larger, sometimes smaller, and often similar effect sizes to inadequately randomized trials. However, they found no average statistically significant difference in effect sizes between the two study types. Yet instead of concluding that adequate randomization had no effect the review authors postulated the “unpredictability paradox”, which states that randomized and non-randomized studies differ, but in an unpredictable direction. However, stipulating the unpredictability paradox is problematic for several reasons: 1) it makes the authors’ conclusion that adequate randomization makes a difference unfalsifiable—if it turned out that adequately randomized trials had significantly different average results from inadequately randomized trials the authors could have pooled the results and concluded that adequate randomization protected against bias; 2) it leaves other authors of reviews with similar results confused about whether or not to pool results (and hence which conclusions to draw); 3) it discourages researchers from investigating the conditions under which adequate randomization over- or under-exaggerates apparent treatment benefits; and 4) it could obscure the relative importance of allocation concealment and blinding which may be more important than adequate randomization.

## Background

Randomization can reduce selection bias and a variety of other confounding factors in healthcare trials [1–4]. We would therefore expect adequately randomized trials to have different results from inadequately randomized trials.

## Main text

In spite of the rationale for adequate randomization, differences between adequately and inadequately randomized trials have proven difficult to detect empirically. In 1995, Schulz and colleagues [1] found that trials using allocation concealment (concealing which participants are in each treatment group) and double-blinding yielded smaller effect sizes, but they found no statistically significant benefit of adequate over inadequate randomization. Odgaard-Jensen and colleagues [5] conducted an overview of systematic reviews in 2011 in an attempt to provide more definitive evidence. The review included systematic reviews comparing randomized trials with trials that used some other, non-random method of assignment to conditions (such as alternation). Of the seven reviews eligible for the meta-analysis, six failed to detect a statistically significant difference between adequately and inadequately randomized trials, and one revealed smaller effects in randomized trials. Three of the six reviews that failed to detect a statistically significant difference suggested that adequate randomization increased effect sizes, and three suggested they reduced effect sizes.

**Figure 1:**
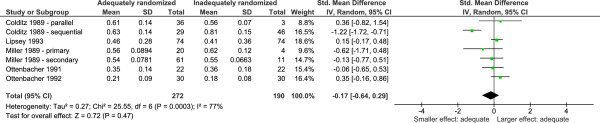
**Pooled results from adequately and inadequately randomized trials in the Odgaard-Jensen and colleagues Cochrane Review** [5]**.** CI, confidence interval; IV, independent variable; RCT, randomized controlled trial; SD, standard deviation; Std, standardized.

Had they pooled the results (which we did, see Figure 1), they would have reported no statistically significant difference between the two study types, yet Odgaard-Jensen and colleagues did not pool the results. Instead they asserted that the results from randomized and non-randomized studies differ, but in an unpredictable direction: “it is not generally possible to predict the magnitude, or even the direction, of possible selection biases and consequent distortions of treatment effects from studies with non-random allocation” [5]. They called this the “unpredictability paradox”.

Yet there are several problems with the inference to the “unpredictability paradox” from the observed data.

1. Invoking the unpredictability paradox makes the conclusions of the Odgaard-Jensen review unfalsifiable and unscientific (from a Popperian perspective) [6]. If it turned out that randomized trials had average significantly different average results from non-randomized studies, the authors could have pooled the results and concluded that adequately randomized trials were better. In fact, adequate randomization did not yield statistically significant different average results, and the authors drew the very same conclusion that they could have had the data indicated differences between adequately and inadequately randomized trials. Drawing the same conclusion from conflicting evidence allows us to make assertions that do not take empirical evidence into account, which is unscientific in the absence of further justification.
2. Appeal to the unpredictability paradox reveals an inconsistent approach with regards to pooling data in Cochrane Review methodology. When we pooled the results from the Odgaard-Jensen and colleagues review we found no statistically significant difference between randomized and non-randomized trials (standardized mean difference = −0.17, 95% CI = −0.64 to 0.29; *P* = 0.47; Figure 1). The decision to pool appears to justify the inference to the conclusion that adequate randomization was not a methodological benefit easy to draw. (As an aside, the problem is not whether to pool itself, but rather the inference from the unpooled result to the conclusion of a difference in an unpredictable direction.) The Cochrane Handbook recommends not pooling highly heterogeneous results [7], yet the results of the Odgaard-Jensen and colleagues review were remarkably consistent in terms of effect direction, with all but one included study revealing no statistically significant difference. Moreover Cochrane Reviews conducted by the same review group have pooled results with substantially higher heterogeneity (I^2^ = 87%) [8]. The inconsistency in Cochrane methodology was further highlighted in a recent similar systematic review of randomized versus observational studies. The authors of the latter review found similarly heterogeneous results, but decided to pool and concluded that randomized and non-randomized studies were not qualitatively different [9]. Had they adopted the same strategy as Odgaard-Jensen and colleagues they could have chosen not to pool, postulated the “unpredictability paradox” and concluded that randomized trials have different results from observational studies, but in an unpredictable direction.
3. The unpredictability paradox has not been used or replicated independently [10]. If proposing that the unpredictability paradox is justified, one would expect independent research to use and validate it. This has not been done.
4. Invoking the unpredictability paradox discourages researchers from investigating the conditions under which randomization over- and under-exaggerates apparent treatment benefits. If, indeed, adequate randomization makes a difference, it would be interesting to know what made adequate randomization increase effect size and what made it decrease effect size. Proposing the unpredictability paradox as an explanation for the effect of adequate randomization suggests that there is nothing more fundamental to be learned about the conditions under which adequate randomization makes a difference, precisely because it is unpredictable. This approach therefore arguably stifles future research in the area.
5. If it turns out that adequate randomization is not a powerful protection against bias, it could obscure the relative importance of allocation concealment and blinding which may be more important.

## Discussion

Our arguments presented here do not imply that inadequate randomization is acceptable. In fact one of us has written a book defending the virtues of (adequate) randomization [11]. We believe it is self-evident that inadequate randomization is a sign of sloppy research, and also makes allocation concealment and blinding more difficult. Allocation concealment and blinding, in turn, have been shown empirically to reduce bias in many cases [4, 12]. It follows that, when results from adequately randomized studies and inadequately randomized studies (or observational studies) differ, the results of the adequately randomized trial is likely to be closer to the truth (all other things being equal).

## Conclusions

Our conclusion is that Odgaard-Jensen and colleagues’ proposed unpredictability paradox requires further justification. Providing a justification will improve the soundness and validity of the Odgaard-Jensen and colleagues review, inform debates about when to pool heterogeneous results in systematic reviews, rationalize Cochrane Review methodology, and tell us more about the mechanism by which adequate randomization reduces bias. Critical appraisal tools [13, 14], and justification for the inclusion of studies in systematic reviews may also need to be revised in light of an eventual justification for the unpredictability paradox.
